# Chlorate of Potash in Croup

**Published:** 1854-03

**Authors:** 


Chlorate of Potash in Croup.—The following facts, as given by Dr. Sankey, corroborate the views laid down by Dr. Budd in his paper on Croup: a child aged thirteen months, with croup and bronchitis, was treated with blisters, salines, ipecac, &c., without benefit, when he was put upon the use of *chlorate of potash;* and although the child was much reduced, the circulation languid, the blood not properly oxygenized, yet in a short time the croup and bronchitis disappeared, and the child was restored to its usual good health.

Another child, æt. three years, with croup, had been treated with leeches, calomel and ipecac, without much benefit; he was put upon the *chlorate of potash*, and soon his breathing became easy, and his countenance lost its anxious and livid hue. It recovered as in the first case. The cholorate of potash is supposed to be in part decomposed, in these cases, and thus a large portion of its oxygen is given to the blood, for the absence of which the child perishes in attacks of croup.—*N. O. Med. Journal.*

